# Skeletal muscle magnetic resonance biomarkers correlate with function and sentinel events in Duchenne muscular dystrophy

**DOI:** 10.1371/journal.pone.0194283

**Published:** 2018-03-19

**Authors:** Alison M. Barnard, Rebecca J. Willcocks, Erika L. Finanger, Michael J. Daniels, William T. Triplett, William D. Rooney, Donovan J. Lott, Sean C. Forbes, Dah-Jyuu Wang, Claudia R. Senesac, Ann T. Harrington, Richard S. Finkel, Barry S. Russman, Barry J. Byrne, Gihan I. Tennekoon, Glenn A. Walter, H. Lee Sweeney, Krista Vandenborne

**Affiliations:** 1 Department of Physical Therapy, University of Florida, Gainesville, FL, United States of America; 2 Departments of Pediatrics and Neurology, Oregon Health & Science University, Portland, OR, United States of America; 3 Department of Statistics, University of Florida, Gainesville, FL, United States of America; 4 Advanced Imaging Research Center, Oregon Health & Science University, Portland, OR, United States of America; 5 Department of Radiology, Division of Neurology, The Children’s Hospital of Philadelphia, Philadelphia, PA, United States of America; 6 The Children’s Hospital of Philadelphia, Philadelphia, PA, United States of America; 7 Nemours Children’s Hospital, Orlando, FL, United States of America; 8 Department of Pediatrics and Molecular Genetics and Microbiology, Powell Gene Therapy Center, University of Florida, Gainesville, FL, United States of America; 9 Department of Physiology and Functional Genomics, University of Florida, Gainesville, FL, United States of America; 10 Department of Pharmacology and Therapeutics, University of Florida, Gainesville, FL, United States of America; Universite de Nantes, FRANCE

## Abstract

**Objective:**

To provide evidence for quantitative magnetic resonance (qMR) biomarkers in Duchenne muscular dystrophy by investigating the relationship between qMR measures of lower extremity muscle pathology and functional endpoints in a large ambulatory cohort using a multicenter study design.

**Methods:**

MR spectroscopy and quantitative imaging were implemented to measure intramuscular fat fraction and the transverse magnetization relaxation time constant (T_2_) in lower extremity muscles of 136 participants with Duchenne muscular dystrophy. Measures were collected at 554 visits over 48 months at one of three imaging sites. Fat fraction was measured in the soleus and vastus lateralis using MR spectroscopy, while T_2_ was assessed using MRI in eight lower extremity muscles. Ambulatory function was measured using the 10m walk/run, climb four stairs, supine to stand, and six minute walk tests.

**Results:**

Significant correlations were found between all qMR and functional measures. Vastus lateralis qMR measures correlated most strongly to functional endpoints (|ρ| = 0.68–0.78), although measures in other rapidly progressing muscles including the biceps femoris (|ρ| = 0.63–0.73) and peroneals (|ρ| = 0.59–0.72) also showed strong correlations. Quantitative MR biomarkers were excellent indicators of loss of functional ability and correlated with qualitative measures of function. A VL FF of 0.40 was an approximate lower threshold of muscle pathology associated with loss of ambulation.

**Discussion:**

Lower extremity qMR biomarkers have a robust relationship to clinically meaningful measures of ambulatory function in Duchenne muscular dystrophy. These results provide strong supporting evidence for qMR biomarkers and set the stage for their potential use as surrogate outcomes in clinical trials.

## Introduction

Duchenne muscular dystrophy (DMD) is a severe, progressive muscle wasting disorder. Encouraging new approaches for treatment are transitioning from preclinical investigation to clinical trials.[1] Therapeutic strategies being investigated clinically include nonsense mutation read-through, exon-skipping, utrophin upregulation, and novel anti-inflammatory approaches.[2] As trials advance, the selection of endpoints is crucial and influences trial duration, sample size, and sensitivity to therapeutic efficacy. However, progress in drug development has outpaced the development of trial endpoints with a recognized dearth of objective, sensitive, and noninvasive outcomes and biomarkers for DMD.[3]

Quantitative magnetic resonance (qMR) measures of muscle pathology are emerging as potentially powerful, noninvasive biomarkers for use in clinical trials for DMD.[4] Muscle fat fraction (FF), which measures fatty infiltration, tracks advanced pathology in DMD and can be quantified using either ^1^H magnetic resonance spectroscopy or chemical shift-based imaging techniques.[5–10] The MRI transverse magnetization relaxation time constant (T_2_) is sensitive to several pathological features of DMD including fat infiltration, muscle damage, and inflammation/edema.[11–13] Both measures of muscle FF and MRI T_2_ are reproducible from day-to-day and between study sites,[14] can differentiate boys with DMD from controls,[7,12,15,16] show sensitivity to disease progression with age and over time,[8,17–19] and demonstrate response to corticosteroid treatment,[20] making them potentially valuable biomarkers to evaluate therapeutic efficacy.

One of the gaps in the establishment of qMR biomarkers in DMD, and its acceptance by regulatory authorities, is a thorough investigation of their relationship with clinically meaningful outcomes.[21–22] Initial studies have demonstrated significant correlations between ambulatory function and lower extremity qMR biomarkers, and a handful have examined the relationship between loss of ability and qMR biomarkers.[11,23–28] However, these relationships have only been investigated in a limited fashion through single-site studies with small cohorts. The *ImagingDMD* cohort, consisting of participants enrolled in a natural history MRI study at three sites in the US, is unique in its size and comprehensive characterization of both functional ability and muscle pathology using qMR measures and is well-suited for a careful analysis of the association between function and MR measures. As such, the objective of this study was to determine the cross-sectional relationship between qMR biomarkers of lower extremity muscle pathology, specifically MRI T_2_ and muscle FF, and functional outcomes in a large, primarily ambulatory cohort of individuals with DMD enrolled in the multicenter *ImagingDMD* study. Second, this study examined the association between qMR measures and sentinel events, including loss of ambulation.

## Methods

### Study design, protocol approvals, and consent

Participants with DMD (baseline ages 4–14 years) enrolled in the longitudinal, multicenter *ImagingDMD* observational study (NCT01484678; imagingdmd.org). Participants had a genetically confirmed diagnosis of DMD and showed clinical symptoms before 5yrs. They were required to walk independently >100m and climb four stairs at study entry. Exclusion criteria included contraindications to MRI, unstable or confounding health issues affecting muscle function, and/or an inability to comply with testing. Beginning September 2010, participants visited the study sites for baseline MR and functional data collection, and participants returned every 12 months for up to four years of follow-up. The Institutional Review Board approved this study at each site (University of Florida, Oregon Health & Science University, and the Children’s Hospital of Philadelphia). Informed written consent was obtained from the parent/guardian prior to participation, and assent was obtained from the participant.

### MR acquisition and analysis

**Fig 1:**
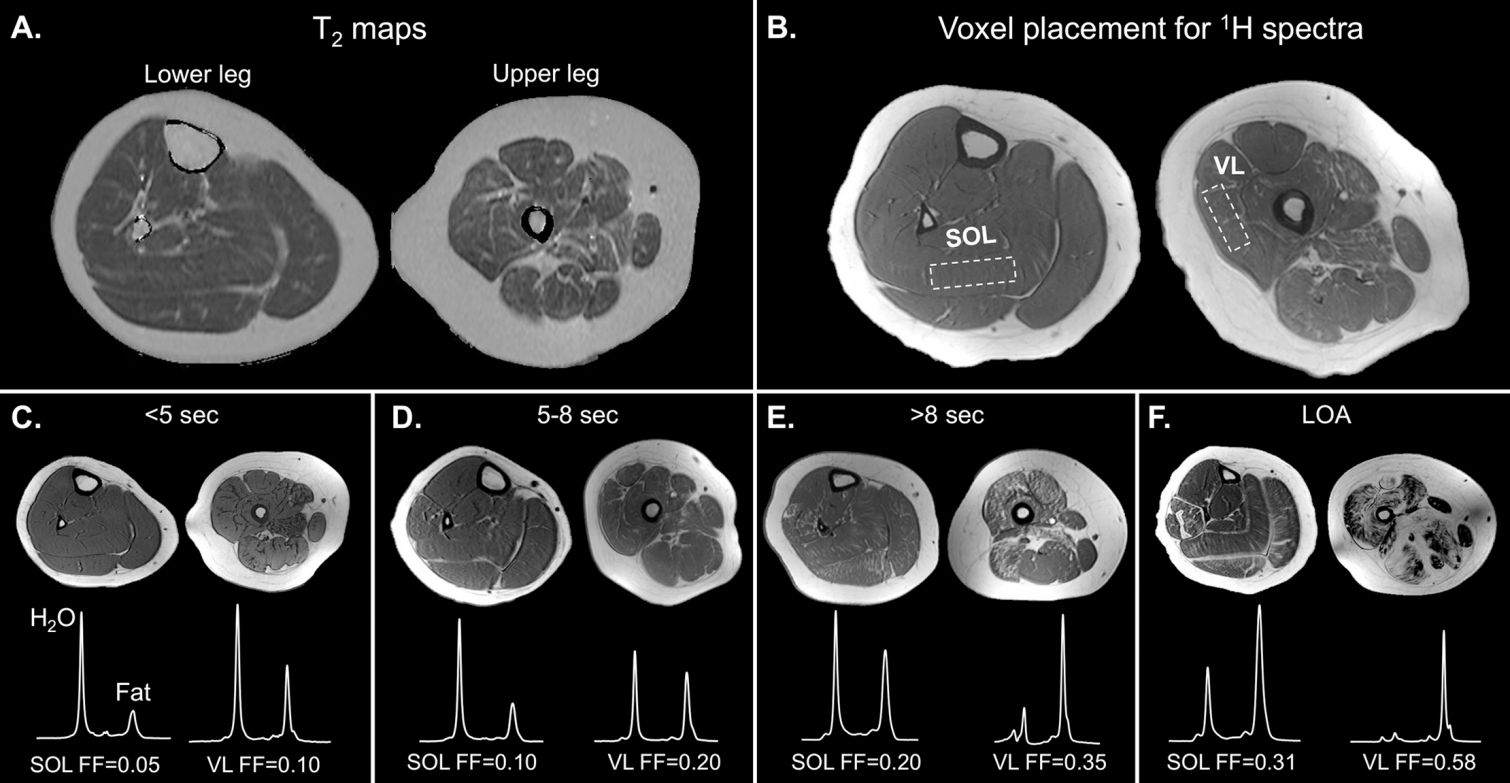
Representative T_2_ maps, ^1^H MRS voxel placement, and ^1^H MR spectra. A) T_2_ maps were created from the 40, 60, 80, and 100ms TE spin echo images of the lower leg and thigh. Utilizing the popliteus and biceps femoris short head as standardized anatomical landmarks to guide lower leg and thigh slice selection, the TA, PER, TP, SOL, MG, VL, BFLH, and GRA muscles were traced on three contiguous slices to determine mean T_2_ for each muscle. B) T_1_-weighted images demonstrate the location of voxel placement for MR spectroscopy used to quantify FF. Voxels were sized as large as possible while staying completely within the SOL and VL muscles, avoiding the fascia. The voxel position from prior years was referenced to position voxels at an anatomically similar location at follow-up visits. C-F) T_1_-weighted images of the calf and thigh with corresponding SOL and VL spectra and FFs from a representative participant in each of four functional groups (based on the 10m walk/run test). Participants have increasing levels of SOL and VL fat infiltration (appreciated visually on the T_1_-weighted images and quantified using spectroscopy) with decreasing ambulatory ability. 10m walk/run times were 3.56s, 7.16s, and 11.32s for the example participants in panels C, D, and E, respectively. (TA = tibialis anterior, PER = peroneus longus and brevis, TP = tibialis posterior SOL = soleus, MG = medial gastrocnemius, VL = vastus lateralis, BFLH = biceps femoris long head, GRA = gracilis, FF = fat fraction).

Prior to initiating the *ImagingDMD* study, each imaging site completed a standardization process using phantoms and a manual of operating procedures. This process, as well as detailed MR data acquisition methods for this study, have previously been described and reproducibility established.[12,14] Briefly, data were acquired using whole-body 3T MRI scanners (Philips Achieva Quasar Dual 3T, Siemens Magnetom Verio 3T, and Siemens Magnetom TIM Trio 3T). Following T_1_-weighted 3D gradient-echo image acquisition, single voxel ^1^H MR spectroscopy was used to quantify intramuscular FF in the vastus lateralis (VL) and soleus (SOL). Spectra were acquired using stimulated echo acquisition mode (STEAM) with TR = 3,000ms, TE = 108ms, 4 phase cycles, and 16 acquisitions, which were averaged after excluding outliers.[14] FF, defined as fat / fat + water signal, was determined using area integration of the phase-corrected averaged spectra, with signal corrected for T_1_ and T_2_ relaxation.[5] MRI T_2_ data were acquired using a T_2_-weighted spin-echo sequence with 7mm slice thickness and TR = 3,000ms. Using a monoexponential decay model, T_2_ maps were created from 40, 60, 80, and 100ms TEs.[12] MRI T_2_ was determined by tracing the medial gastrocnemius (MG), SOL, tibialis anterior (TA), tibialis posterior (TP), peroneal group (peroneus longus and peroneus brevis—PER), VL, biceps femoris long head (BFLH), and gracilis (GRA) on T_2_ maps from three contiguous slices. For each muscle, the reported T_2_ is the mean T_2_ from the three slices. MRI muscle tracing was performed at a single institution (University of Florida) by analyzers who underwent a standardized training and certification process. Fig 1 shows examples of T_2_ maps, MR spectroscopy voxel placement, and ^1^H spectra from participants with different degrees of muscle pathology.

### Functional data collection

After MR data acquisition, participants performed the supine to stand (STS), 10m walk/run, four stair climb, and six minute walk tests (6MWT), which are commonly used in natural history studies and clinical trials.[29–31] For the first three timed tests, up to three trials were performed, and the fastest time to complete the task was recorded. If a participant was unable to complete the task within 45 seconds or without assistance, the participant was considered to have lost the ability to perform that test. Loss of ambulation is defined here as the inability to perform the 10m walk/run. Qualitative grades (one to six) were used to describe how the participant performed the timed tests.[32] The 6MWT was completed on a 25 meter course, and the distance traversed in six minutes was recorded. Instructions and test administration were standardized, and the 6MWT protocol was adapted from that published specifically for DMD.[33]

### Statistical analysis

MR and functional data underwent quality assurance by one or more expert reviewers. Missing and invalid data were excluded from analysis (9.8% for MRI T_2_, 5.5% for MRS FF, and 6.6% for functional tests). Reasons for invalid MR data included artifacts due to excessive motion and signal inhomogeneity. Statistical analyses were performed using R version 3.2.3. For all analyses, participant data were included from the initial visit as well as any subsequent visits in which the participant retained the ability to complete the functional task of interest. Additionally, the first visit in which a participant could no long complete a functional task was included. Therefore, participants who participated across multiple years had MR and functional data from multiple visits included. Although all analyses are cross-sectional in nature, inclusion of data from multiple years of participation allowed for analysis of a more comprehensive data set and for analysis of sentinel events. Data were analyzed using nonparametric statistics due to not meeting criteria such as independence of samples. To appropriately quantify uncertainty (confidence intervals), a nonparametric bootstrap of data with resampling of participants, not individual data points, was used with 1000 samples.[34] Statistical significance was determined by the 95% bootstrap confidence interval excluding zero.

The nonparametric Spearman’s rank correlation coefficient, ρ, was computed to describe the correlation between the qMR and functional measures. For correlation analyses, participants who lost the ability to perform the timed tests were given the maximum time allowed, 45 seconds, which is appropriate for the rank-based Spearman’s correlation. Only data from the first visit in which a participant lost ability were included in the correlation analyses. For pairwise differences of MR measures between functional groups, the bootstrap confidence intervals were adjusted with a Bonferroni correction. To determine the ability of MR values to discriminate between ambulatory and nonambulatory individuals, the area under the receiver operating characteristic (ROC) curve was calculated, again with bootstrap confidence intervals.

## Results

### Cohort characteristics

At the time of analysis, the *ImagingDMD* cohort consisted of 136 participants with DMD. Two enrolled participants were unable to complete assessments at their initial visit and discontinued the study. Cohort characteristics are presented in Table 1. Overall, the *ImagingDMD* cohort studied was young and highly functional at the initial visit with a mean age of 8.3 years (range 4.8–14.6) and with all participants able to ambulate and climb stairs per inclusion criteria. On average, participants completed the timed tests in six seconds or less and walked more than 350m on the 6MWT. Additionally, upon study entry, the mean VL FF of the cohort was below 0.2 and mean VL T_2_ was <50 ms (min = 35.4 ms).

**Table 1:** Cohort characteristics and demographics.

**Demographics at Initial Visit**	
Number of participants	n = 136
Age (years)	8.3 ± 2.2
Height (cm)^a^	120.1 ± 10.2
Weight (kg)^b^	28.0 ± 9.7
Steroid use (y/n)^c^	99/35
**Cohort characteristics**	
12 month visit	n = 124
24 month visit	n = 112
36 month visit	n = 98
48 month visit	n = 84
Mean age across all visits	10.1 ± 2.6
Loss of supine to stand	n = 61
Loss of 4 stair climb	n = 48
Loss of ambulation	n = 35

Data are reported as mean ± SD when appropriate. Sample sizes indicate the total number of participants at each time point.

^a^Baseline height was available for n = 132.

^b^Baseline weight was available for n = 131.

^c^Baseline steroid status unknown in two participants.

In the cohort studied, participants averaged >four longitudinal visits, and 84 individuals had a total of five visits resulting in 554 visits in the *ImagingDMD* database. Over the course of the visits, 35 participants lost ambulation, while 48 and 61 lost the ability to climb stairs and complete STS, respectively. Considering data from all visits, muscle FF and MRI T_2_ values generally increased from year to year, with FF and T_2_ from proximal muscles such as the VL and BFLH increasing more than that of distal muscles. Quantitative MR data at baseline and 12 months follow-up from a large subgroup of this cohort (n = 109) have been recently presented in detail,[18] and a comprehensive analysis of all 48 months of longitudinal data in this cohort will be presented at completion of the longitudinal study.

### Cross-sectional relationship between qMR measures and functional endpoints

**Fig 2:**
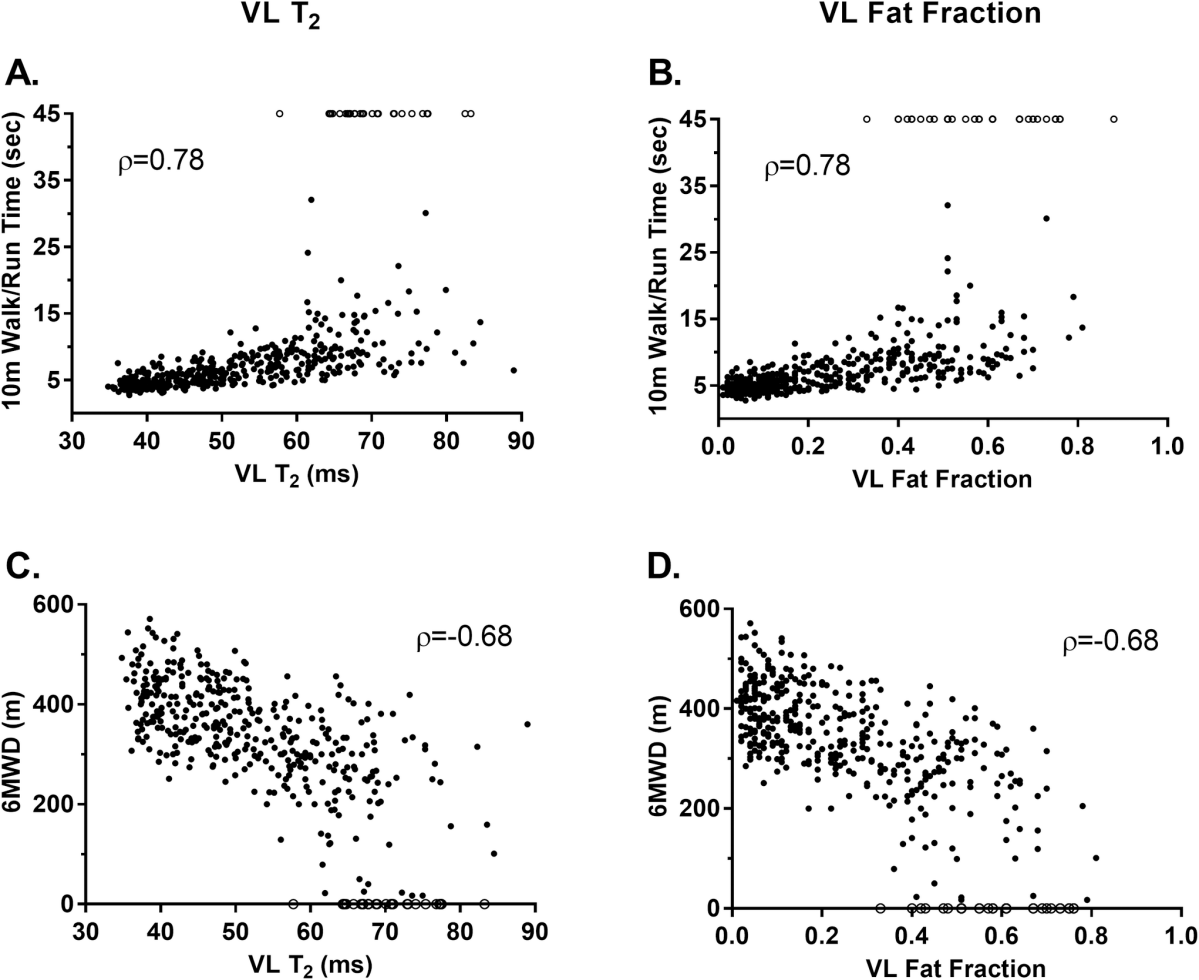
Scatterplots showing the correlations between VL MRI T_2_ and FF and functional endpoints. The qMR biomarkers most strongly correlated to function were VL MRI T_2_ and VL FF. A) VL MRI T_2_ versus 10m walk/run, B) VL FF versus 10m walk/run, C) VL MRI T_2_ versus 6MWD, D) VL FF vs 6MWD. Participants who lost the ability to perform the timed tests were given the maximum time of 45 seconds (open circles) for the first visit in which they were unable to complete the test. Participants who lost the ability to perform the 6 MWT have distances of 0 m (open circles). As indicated in Table 2, all correlations were significant with the 95% bootstrap confidence intervals excluding zero. (VL = vastus lateralis, 6MWD = six minute walk distance, 6MWT = six minute walk test).

Based on data acquired at all participant visits with both MR and functional data, significant correlations were found between qMR measures of muscle pathology in all lower extremity muscles and every ambulatory functional test (Table 2). Among MRI T_2_ measures, the strongest correlations to functional performance were found for the VL, BFLH, and PER, with correlation coefficient values ranging from 0.70 to 0.78 for timed tests and -0.59 to -0.68 for the 6MWT. Based on MR spectroscopy, VL FF (|ρ| = 0.68–0.78) was more strongly related to functional endpoints than SOL FF (|ρ| = 0.58–0.66). VL FF and MRI T_2_ correlations with functional measures were very similar. Globally, MR measures correlated more strongly with the STS, 10m walk/run, and four stair climb tests than the 6MWT (Fig 2).

**Table 2:** Correlation between MRI T_2_, muscle FF, and functional endpoints.

			STS	10m walk/run	4 stair climb	6MWT
**MRI T**_**2**_	**Upper** **Leg**	**VL**	**0.77**	**0.78**	**0.77**	**-0.68**
			(0.71–0.82)	(0.74–0.83)	(0.72–0.81)	(-0.60– -0.74)
			n = 376	n = 445	n = 438	n = 414
		**BFLH**	**0.73**	**0.73**	**0.73**	**-0.63**
			(0.65–0.80)	(0.66–0.79)	(0.66–0.79)	(-0.54– -0.71)
			n = 374	n = 444	n = 438	n = 414
		**GRA**	**0.26**	**0.39**	**0.40**	**-0.30**
			(0.09–0.41)	(0.25–0.52)	(0.25–0.52)	(-0.16– -0.42)
			n = 373	n = 443	n = 436	n = 412
	**Lower** **Leg**	**PER**	**0.70**	**0.72**	**0.72**	**-0.59**
			(0.62–0.77)	(0.65–0.78)	(0.65–0.78)	(-0.50– -0.67)
			n = 379	n = 450	n = 444	n = 423
		**SOL**	**0.62**	**0.68**	**0.67**	**-0.59**
			(0.51–0.72)	(0.60–0.75)	(0.58–0.75)	(-0.49– -0.68)
			n = 388	n = 463	n = 455	n = 434
		**TA**	**0.60**	**0.63**	**0.64**	**-0.54**
			(0.51–0.68)	(0.53–0.70)	(0.55–0.71)	(-0.43– -0.62)
			n = 381	n = 452	n = 445	n = 424
		**MG**	**0.55**	**0.64**	**0.64**	**-0.54**
			(0.44–0.65)	(0.55–0.72)	(0.54–0.72)	(-0.43– -0.63)
			n = 387	n = 462	n = 454	n = 435
		**TP**	**0.39**	**0.49**	**0.46**	**-0.40**
			(0.27–0.49)	(0.38–0.57)	(0.36–0.56)	(-0.28– -0.50)
			n = 379	n = 453	n = 445	n = 425
**MRS FF**		**VL**	**0.77**	**0.78**	**0.78**	**-0.68**
			(0.71–0.82)	(0.73–0.83)	(0.72–0.82)	(-0.60– -0.75)
			n = 387	n = 461	n = 452	n = 432
		**SOL**	**0.60**	**0.66**	**0.65**	**-0.58**
			(0.49–0.69)	(0.57–0.73)	(0.57–0.73)	(-0.49– -0.67)
			n = 404	n = 478	n = 469	n = 446

The correlation between each muscle MR measure and functional test is represented by the correlation coefficient, ρ, and the 95% bootstrap confidence intervals are given in parentheses. N = the number of XY pairs for each given combination. All correlations were significant with the 95% bootstrap confidence intervals excluding zero for all comparisons. Cells are shaded according to the strength of the correlation with higher correlation coefficients shaded in darker gray.

### Comparison of FF across functional groups by ambulatory ability

**Fig 3:**
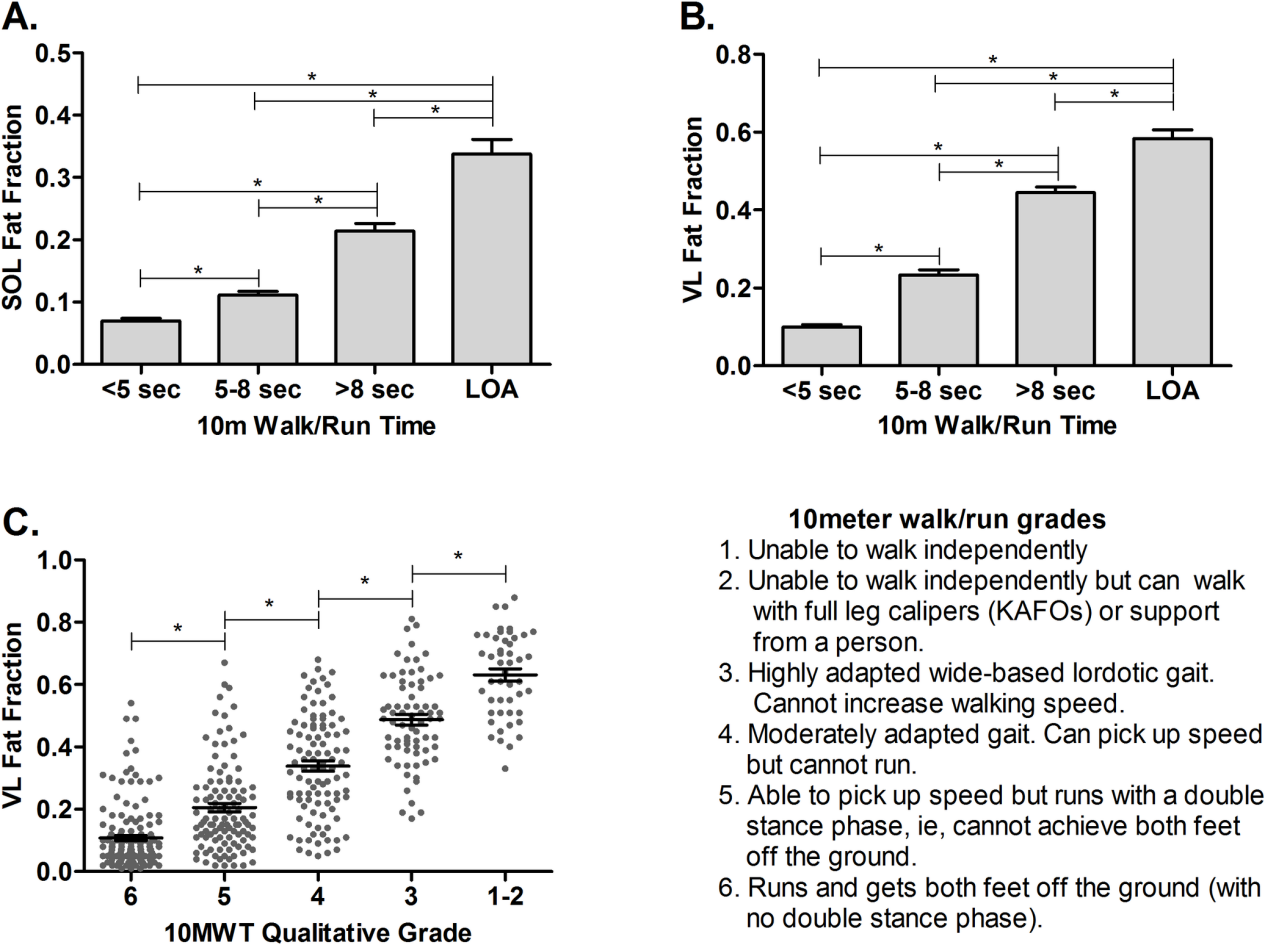
FF and quantitative and qualitative measures of walking/running ability. A and B) FF was assessed based on 10m walk/run performance. Data points from participants who could complete the test were divided into bins of <5 sec (n = 152), 5–8 sec (n = 184), and >8 sec (n = 127). The LOA group represents data points from subjects that could no longer complete the 10m walk/run at the time of the visit (n = 35). The amount of SOL and VL fatty infiltration was significantly different between functional performance groups. C) Groups were created based on the qualitative rating of participants’ 10m walk/run test at each time point. Mean VL FF was significantly different between each qualitative grade group. * = significantly different; bootstrap confidence intervals excluded zero. (SOL = soleus, VL = vastus lateralis).

The link between muscle FF and walking/running ability was investigated in further detail, using both quantitative and qualitative assessments. For the 10m walk/run, data were grouped based on participants’ performance at the time of the visit. 10m walk/run times from participants who were still ambulatory at a specific visit were divided into bins of <5 seconds, 5–8 seconds, and >8 seconds. A fourth group (LOA) was comprised of data points from participants who had lost ambulation at the time of the visit. There were significant differences in both SOL and VL FF between each of the four functional bins, with the highest FF values in the LOA group (Fig 3A and 3B). Similar results were found using a qualitative rating of 10m walk/run ability, based on the Eagle scoring system.[32] As the qualitative rating of walk/run performance decreased, there was a significant increase in VL FF (Fig 3C). T_1_-weighted MR images and ^1^H spectra from a representative participant in each 10m walk/run bin illustrate the progressive increase in muscle fatty infiltration with declining ambulatory ability (Fig 1C–1F).

### qMR biomarkers and loss of functional ability

**Fig 4:**
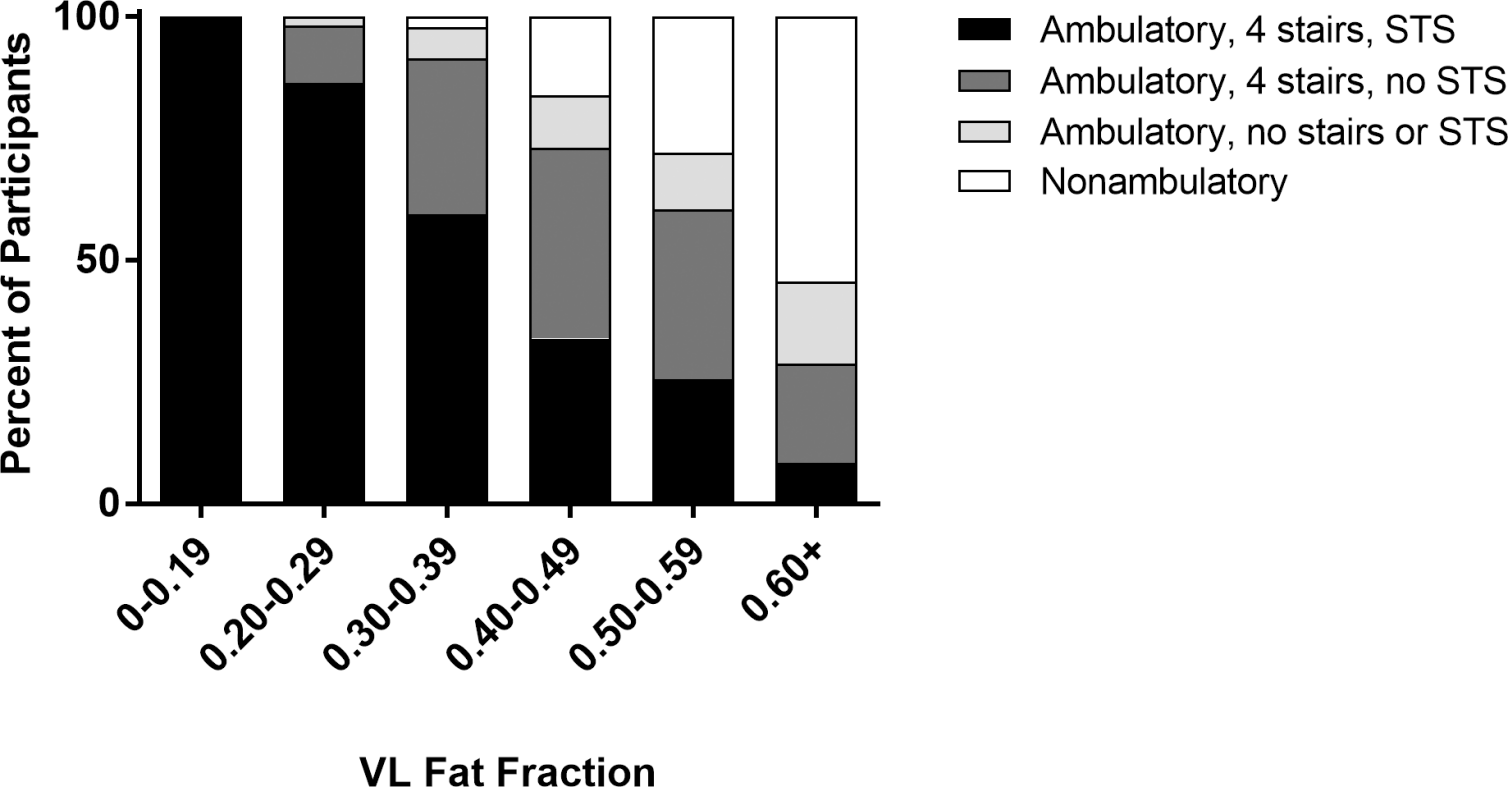
Loss of function in participants with different VL FFs. Data points were binned by VL FF and further divided by the number of functional skills the participants could complete at the time of the visit. Black = ambulatory, able to climb 4 stairs, able to move supine to stand. Dark gray = ambulatory, able to climb 4 stairs, unable to rise from supine. Light gray = ambulatory, unable to perform stairs, unable to rise from supine. White = nonambulatory. As FF increased, there was a progressive decrease in the percent of participants who could perform the functional tasks. At VL FFs less than 0.19, 99% of participants could perform all functional tests. Apart from one outlier, participants began losing ambulation at VL FFs ≥ 0.40. (VL = vastus lateralis, STS = supine to stand).

With increasing VL FF, an increasing percentage of participants demonstrated loss of functional abilities (Fig 4). At low VL FF values (0.01–0.19), 99% of participants were able to perform all three timed functional tests, and even at VL FFs between 0.20 and 0.30, most individuals remained quite functional. Once VL FF was ≥0.60, over 50% of participants were nonambulatory and another 40% had lost the ability to perform either or both the STS and the four stair climb tests. Of the participants who remained ambulatory with a VL FF ≥0.60, STS time averaged greater than >12 sec, stair climb time averaged >9 sec, and 10m walk/run time averaged >11 sec, demonstrating significantly diminished functional performance.

**Fig 5:**
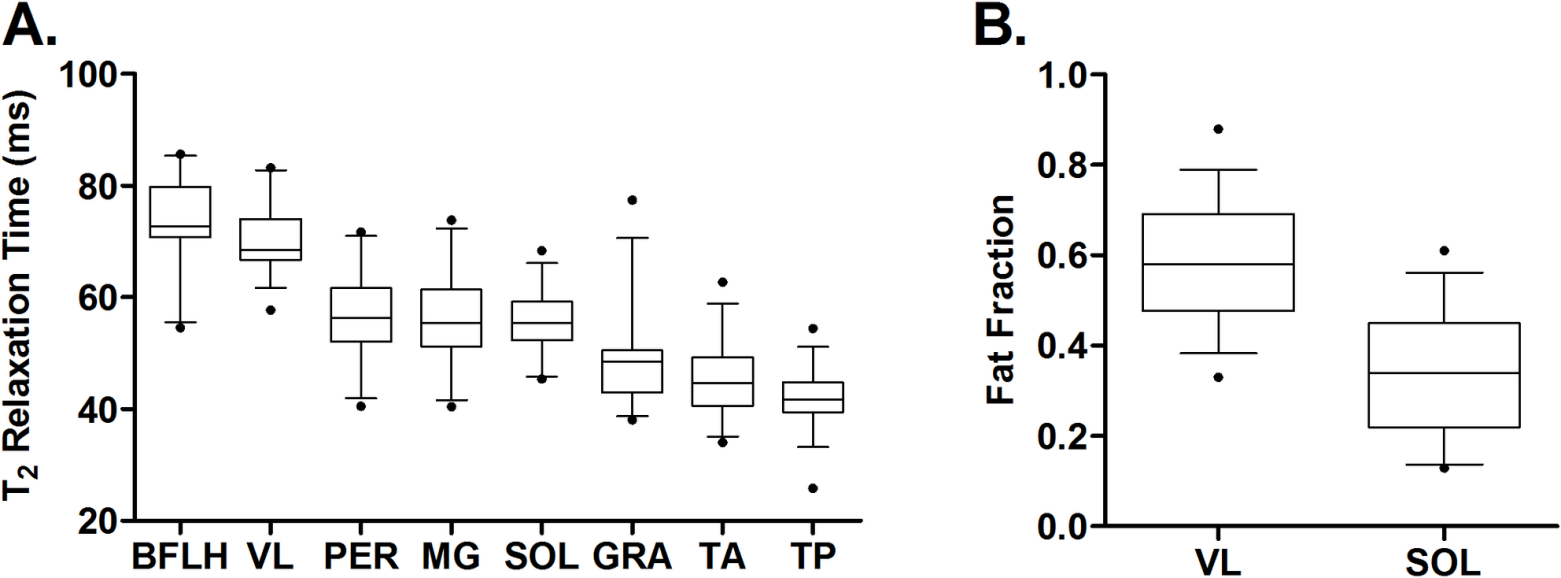
qMR biomarkers of muscle pathology at the first visit following loss of ambulation. A) MRI T_2_ and B) MRS FF are plotted for each muscle at the first time point at which participants were nonambulatory (n = 35). Whiskers represent the 5^th^-95^th^ percentiles with outliers represented by the filled dots. MRI T_2_ was highest in the BFLH and VL and lowest in the GRA, TA, and TP. The MRI T_2_ and FF of the VL were higher than that of the SOL as expected based on the proximal to distal pattern of involvement in DMD.

The mean VL FF at the first visit in which participants could not complete STS was 0.45 (25^th^-75^th^ percentile = 0.38–0.54), while VL FF at the first visit in which they could not climb four stairs was 0.54 (25^th^-75^th^ percentile = 0.43–0.64). Mean VL FF at the first visit following loss of ambulation was highest at 0.58 (25^th^-75^th^ percentile = 0.48–0.69). VL T_2_ values followed the same trend, with T_2_ at the first visit following loss of ambulation occurring at the highest T_2_ value (mean = 70.1 ms; 25^th^-75^th^ percentile = 66.8–74.0 ms). These observations parallel the typical sequence of functional loss seen in the clinic. The status of the lower extremity musculature at the first nonambulatory visit is presented in Fig 5. Of note, apart from one outlier who experienced a fracture that precipitated loss of ambulation, VL FF was never less than 0.40 and VL T_2_ was never less than 60ms in nonambulatory participants An ROC curve of the relationship between ambulation status and VL FF showed an area under the curve of 0.91 (95% CI = 0.87–0.94), demonstrating the ability of VL FF to strongly discriminate between ambulatory and nonambulatory individuals.

## Discussion

In DMD, the paucity of sensitive, noninvasive outcome measures for use in clinical trials has stimulated the development of qMR biomarkers of muscle pathology. This investigation represents the largest cohort study examining the relationship between qMR measures and clinically meaningful functional endpoints in DMD, a prerequisite for demonstrating the utility of MR biomarkers as surrogate outcomes in clinical trials. Using the multicenter *ImagingDMD* cohort, we demonstrated that 1) lower extremity qMR biomarkers, specifically FF and MRI T_2_, strongly correlate to ambulatory functional endpoints; 2) the strongest correlations are found with qMR measures in rapidly progressing muscles (VL, BFLH, PER); and 3) qMR biomarkers, particularly VL FF, are strongly associated with the loss of functional ability and can discriminate between ambulatory and non-ambulatory individuals.

Development of pharmacological therapies for DMD has advanced more rapidly than that of sensitive, noninvasive outcome measures to detect therapeutic efficacy in patients. Clinical outcome measures capture meaningful changes in functional ability but are not well-suited for functionally heterogeneous cohorts and may require impractically long trials to determine efficacy if used exclusively.[29,35–37] Functional outcomes such as the 6MWT have also been criticized for their dependence on motivation, especially in younger populations. Therefore, tremendous efforts have been devoted to developing sensitive biomarkers, including MR biomarkers.

At this time, qMR measures of lower extremity muscles have shown promise as biomarkers and potential surrogate outcomes in DMD. Quantitative MR is objective, sensitive, and noninvasive, and thus well-suited for clinical trials requiring longitudinal measures in a pediatric population. A surrogate endpoint is a biomarker where a change in the biomarker is “expected to reflect changes in a clinically meaningful endpoint.”[22] This study was performed to establish the relationship between MRI T_2_ and MRS FF and meaningful measures of ambulatory function as an important step towards establishing qMR as a surrogate endpoint. Demonstration of a strong correlation between qMR biomarkers and function, as shown in this large, multicenter investigation is necessary. However, these current promising results must be followed by an investigation of the ability of MR biomarkers to predict change in function under natural history and therapeutic conditions.

In our primarily ambulatory cohort, qMR biomarkers from the most quickly progressing muscles, the VL and the BFLH, demonstrated the strongest correlations with all functional endpoints. Both MRI T_2_ and MRS FF were measured in the VL, and correlations with function were similar for both biomarkers. MRI T_2_ is heavily influenced by muscle fatty infiltration, particularly in advanced disease, and closely correlates with FF over the course of disease progression, which is consistent with the similar correlations.[7] In agreement with prior reports, PER T_2_ also showed a strong correlation to ambulatory function.[38] MRI T_2_ from the GRA and TP muscles, which are relatively spared, showed the weakest correlation to ambulatory functional endpoints.[25]

Prior studies in smaller cohorts have likewise found high degrees of correlation between qMR measures from quickly progressing proximal muscles and functional outcomes.[11–12,23–26,28,38] In one study (n = 20), quadriceps muscle FF was highly correlated to the D1 dimension of the motor function measure, even more so than hamstring or adductor FF.[23] Additionally, two studies (n = 20, n = 34) showed a significant relationship between the mean FF or T_2_ of upper leg and pelvic girdle muscles and timed supine to stand and the 10m walk/run.[11,25] A more recent study (n = 13) found that VL FF, measured by IDEAL-CPMG, was highly correlated to 6MWT distance.[28] These smaller studies provided preliminary support for the correlation between qMR measures and functional ability, and our study confirms these findings in a large multicenter cohort.

The relationship between qMR biomarkers and functional ability was strong (VL |ρ| = 0.68–0.78), but as anticipated, the correlation was not perfect for several reasons. First, individuals with DMD use remarkable compensatory mechanisms to maintain the ability to walk and perform functional tests, reducing the direct dependence of functional performance on muscle quality in a single muscle.[39] Second, younger individuals with DMD frequently demonstrate improvement on ambulatory functional performance tests before stabilizing or declining, due to maturation and growth.[36] In contrast, qMR measures of muscle pathology display progressive decline (in the absence of therapeutic intervention) even when functional measures are improving or stable.[18] Thus, estimating the relationship between MR biomarkers and function as linear may be overly simplistic. Finally, qMR biomarkers demonstrate superior sensitivity to disease progression compared to measures of function, which impacts the correlation between the two.[18,28]

Sentinel events such as loss of ambulation are important milestones in the progression of DMD and are used as endpoints in clinical trials and natural history studies. Loss of the ability to perform functional skills was strongly associated with fatty infiltration of the proximal lower extremity musculature, particularly the VL muscle. Apart from one outlier, in our cohort, no nonambulatory participants had a VL FF<0.40, indicating that this might be an approximate lower threshold of muscle pathology associated with loss of ambulation. Using chemical shift-based imaging techniques, previous studies found similar relationships between proximal muscle FF and loss of functional ability. Gaeta *et al*. found that boys who lost the ability to complete STS had a gluteus maximus FF >75%, and Fischmann *et al*. reported that loss of ambulation was associated with a mean FF of >50% for the quadriceps, hamstrings, and adductors combined.[24,25] Taken in combination with results from our investigation in the *ImagingDMD* cohort, there is strong evidence that loss of ambulatory skills is closely related to the degree of proximal lower extremity muscle fat infiltration.

The robust relationship between qMR biomarkers and ambulatory function provides further evidence that qMR biomarkers, when implemented carefully, satisfy requirements for surrogate outcome status and should receive serious consideration for inclusion in clinical trials. These biomarkers are reproducible across multiple centers,[14] highly sensitive to disease progression,[18] responsive to corticosteroid therapy within 3–6 months,[20] and, as shown here, strongly correlated to commonly used clinical measures of function. Importantly, they are also reflective of key characteristics of the disease pathology. Building off the work presented here and in prior studies, the next step should be an examination of qMR biomarkers longitudinally and their ability to predict change in, or loss of, function. Using qMR biomarkers as surrogate outcomes in clinical trials can lead to shorter trials requiring fewer participants than trials using traditional clinical outcomes. In fact, power analyses reveal that using qMR biomarkers rather than the 6MWT or motor function measure can reduce the number of participants needed in a trial by well over half.[18,23] Reduced trial times and fewer participants can lead to lower costs and more rapid progress in drug development for this life-limiting disease. Results from longitudinal studies of other neuromuscular diseases including Charcot-Marie-Tooth, inclusion body myositis, limb girdle muscular dystrophy, and facioscapulohumeral muscular dystrophy have likewise demonstrated the utility and sensitivity of qMR biomarkers as potential surrogate outcomes.[40–42]

Selection of the most appropriate qMR biomarkers or muscles to be included in future clinical trials will depend on the specific cohort (age, functional status) and the goal of the therapeutic intervention. In the current study, qMR biomarkers of the VL and BFLH muscles correlated most strongly with both measures of ambulatory function and loss of ambulation. In our experience, qMR biomarkers from the VL offer additional advantages including the muscle’s clear functional role as a major leg extensor, its sensitivity to change over time, and its large size, which allows for excellent sampling of the muscle by MR spectroscopy or by manual segmentation.[18] Thus, we recommend that future trials including ambulatory participants consider the inclusion of VL qMR biomarkers.

A limitation of this study was that the analysis of loss of functional ability was somewhat restricted by inclusion criteria (ability to walk >100m and climb four stairs) and the small number of individuals who lost the ability to ambulate (n = 35) or climb stairs (n = 48) in this cohort. Additionally, although not a limitation, we elected to implement ^1^H MR spectroscopy rather than the popular Dixon imaging technique to quantify muscle FF. While each technique has strengths and limitations, MR spectroscopy, the gold standard for muscle fat quantification, is highly sensitive to disease progression and less susceptible to noise bias at low FFs. Finally, future investigations should explore the concept of composite qMR measures, which may be superior to individual muscle measures, but alternately may increase measure variability, decrease sensitivity, or obscure the relationship between measure change and clinical benefit.[38]

Clinical trial progress in DMD has been impeded by a lack of sensitive, noninvasive, and objective outcomes and biomarkers suited for longitudinal studies in a pediatric population. Quantitative MR measures have emerged as valuable biomarkers to fill this void. One gap in the establishment of MR biomarkers has been a robust demonstration of the correlation with clinically meaningful measures of function and sentinel events. The results of this study, building upon the promising results from other groups with smaller cohorts, convincingly support the link between function and qMR biomarkers. Studies in other neuromuscular diseases have also demonstrated responsiveness of MR measures, highlighting the applicability of qMR biomarkers not only to DMD but potentially to a wider range of progressive neuromuscular disorders.[40–42]
